# Supplementary material for: Functional and Kinetic Comparison of Alanine Cysteine Serine Transporters ASCT1 and ASCT2
Source: Biomolecules. 2022 Jan 11;12(1):113. doi: 10.3390/biom12010113 (PMC8773628; doi:10.3390/biom12010113)
Supplement: Supplementary file 1 [file biomolecules-12-00113-s001.zip › biomolecules-1536175-supplementary.pdf]

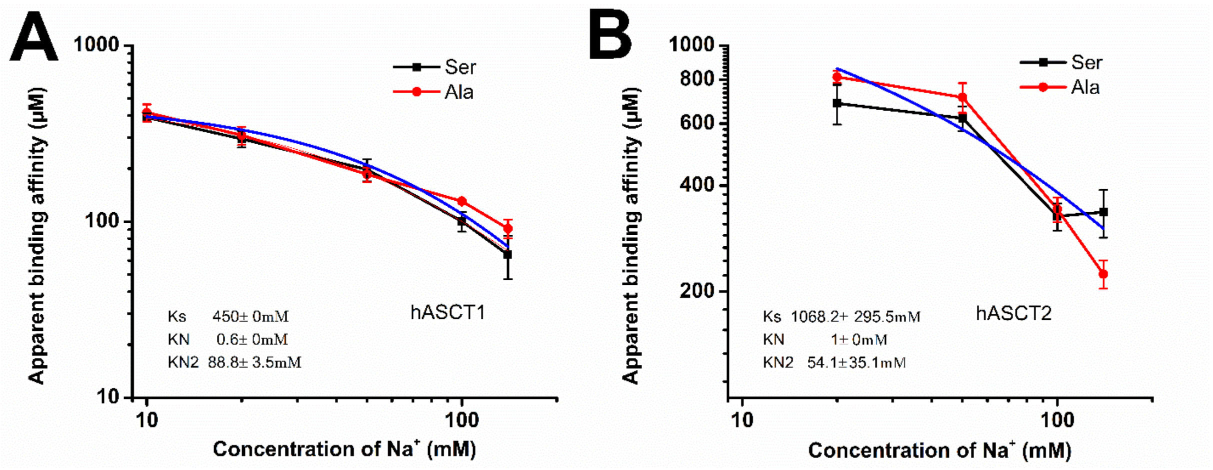

Suppl. Figure S1: Apparent affinity for substrate is dependent on [Na$^+$].

Apparent K_m values for the substrates serine and alanine affinities for hASCT1 **(A)** and 2 **(B)** plotted as a function of extracellular [Na$^+$]. Intracellular solutions contained 130 mM NaSCN with 10 mM of the same substrate, The black line represents results for serine, the red line for alanine. The blue line shows the result from a fit to the following equation: $K\text{m} = K\text{s} * \{[(K_N + [\text{Na}^+])/[\text{Na}^+]^2] * [K_{N2/} K_{N2} + [\text{Na}^+]\}$.

hASCT1: Ks: 450 µM, K_N: 0.6 mM, K_{N2}: 89 mM.

hASCT2: Ks:1070 µM, K_N: 1 mM, K_{N2}: 54 mM.

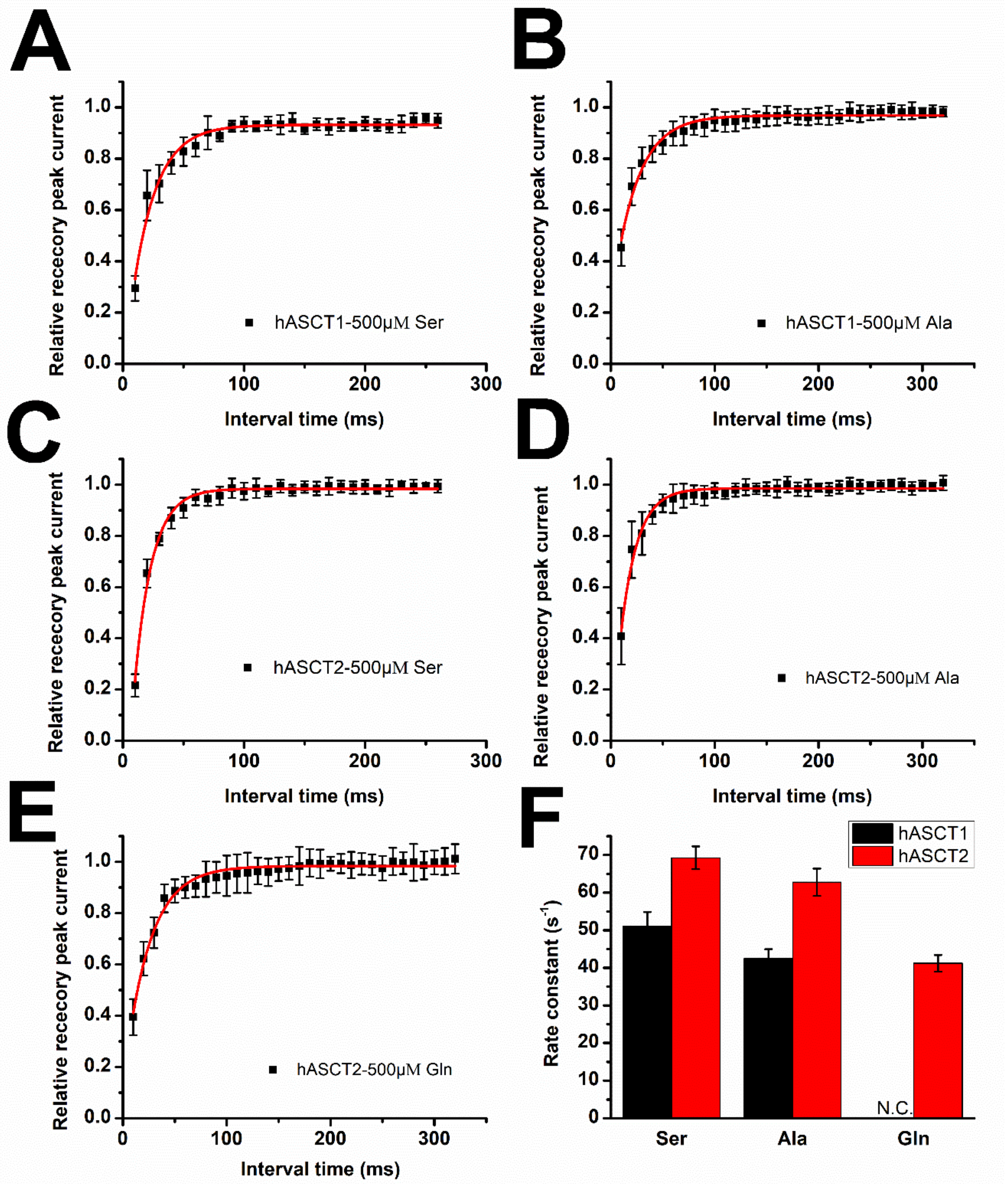

Suppl. Figure S2: Transient current recovery rates depend on the substrate

Representative results from fitting the recovery of peak transient currents after substrate removal for various substrates **(A, C)** serine, **(B, D)** alanine, **(E)** glutamine. Fitting methods were the same as shown in Fig.6. Each amino acid substrate was used on both sides of the membrane (10 mM internal concentration) **(F)** Comparison of recovery rate constants for ASCT1 and 2.

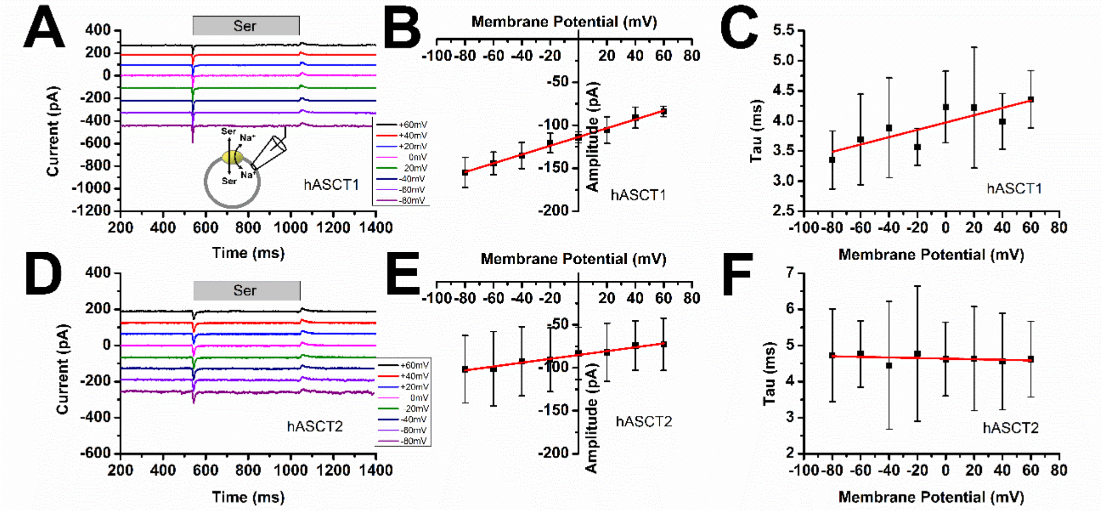

Suppl. Figure S3: Substrate-induced transient currents show low voltage dependence

Transient currents in response to rapid solution exchange (application and removal) were tested as a function of the membrane potential. Typical 500 µM serine-evoked transient currents are shown in **(A)** for ASCT1 and **(D)** for ASCT2. **(B)** and **(E)** Voltage dependence of the peak amplitude of transient currents. **(C)** and **(F)** Time constant of the current decay after serine application as a function of the voltage.
